# Two-dimensional W_8_S_6_Se_6_ monolayers: intrinsic semiconductors with high anisotropic carrier mobility and optoelectronic performance from first-principles

**DOI:** 10.1039/d6ra03466g

**Published:** 2026-09-21

**Authors:** Yi Peng, Minjia Yao, Fangyuan Li, Yu Chen, Yana Li, Shuangmei Qian, Qianqian Zhu, Juexian Cao

**Affiliations:** a Microelectronics and Optoelectronics Technology Key Laboratory of Hunan Higher Education, School of Physics and Electronic Electrical Engineering, Xiangnan University Chenzhou 423000 P. R. China; b Hunan Institute of Advanced Sensing and Information Technology, Xiangtan University Xiangtan 411105 PR China pengzhu2022@yeah.net

## Abstract

Two-dimensional (2D) semiconductors hold tremendous promise for the development of next-generation optoelectronics and electronics. Nonetheless, existing 2D semiconductors are plagued by inherently low carrier mobility at ambient temperatures, which restricts their possible applications. In this work, we explore several crystalline phases of W_8_S_6_Se_6_ monolayers, assessing their structural stability and fundamental characteristics through first-principles calculations. Our results demonstrate that all the investigated structures maintain remarkable thermodynamic and mechanical stability. Electronic structure analysis indicates that *P*6*m*2, *P*6*mm*, *P*3*m*1, *Cmm*2, and *C*2/*m*-W_8_S_6_Se_6_ monolayers function as intrinsic semiconductors, exhibiting bandgaps between 1.01 and 1.04 eV, according to the HSE06 functional level. Additionally, these monolayers showcase impressive ductility and exhibit excellent light absorption coefficients of 10^5^ cm^−1^ across the near-infrared to visible spectrum. Remarkably, they display considerable anisotropic carrier mobility, with electron mobility in the *y* direction frequently reaching 10^4^ cm^2^ V^−1^ s^−1^. Overall, these innovative W_8_S_6_Se_6_ monolayers not only expand the repertoire of 2D optoelectronic materials but also offer promising avenues for future nanoelectronics and sensors.

## Abstract 2

These W_8_S_6_Se_6_ monolayers show high electron mobilities (up to 104 cm^2^ V^−1^ S^−1^) along *y* direction, two orders higher than Janus WSSe monolayers. W_8_S_6_Se_6_ monolayers are promising for high-performance optoelectronic devices.

## Graphical abstract

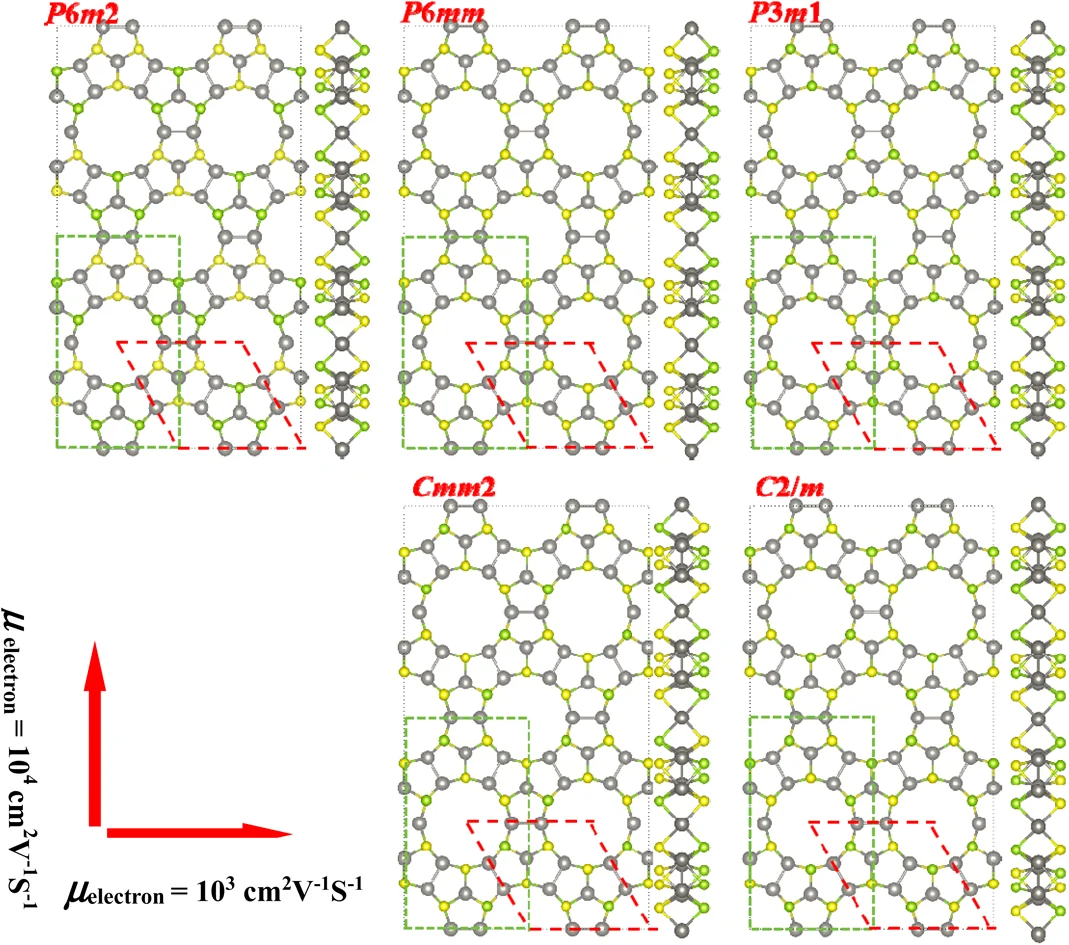

## 1. Introduction

In the past decade, 2D optoelectronic materials have garnered considerable research interest due to their adjustable band structures, superior electron transport capabilities, and substantial exciton binding energies.^1–5^ It is widely recognized that exceptional 2D optoelectronic materials possess not only an appropriate direct bandgap but also remarkably high mobility.^2,6,7^ Consequently, the pursuit of innovative 2D materials that exhibit impressive mobility and a favorable direct bandgap is motivated by both fundamental scientific curiosity and the pressing demand for next-generation optoelectronic applications.

To date, substantial advancements have been achieved in the synthesis of ultrathin 2D materials, including graphene,^8,9^ phosphorene,^10,11^ and transition metal dichalcogenides (TMDs).^12,13^ For these materials to perform effectively in high-performance field-effect transistors (FETs), they require a moderate direct bandgap coupled with elevated charge carrier mobility. Nonetheless, each of these existing alternatives comes with its own distinct set of challenges. While graphene is celebrated for its outstanding carrier mobility (∼10^5^ cm^2^ V^−1^ s^−1^ at room temperature), it is constrained by an intrinsic zero band gap that can only be weakly tuned *via* strain or nanostructuring.^14,15^ Conversely, phosphorene boasts an ideal direct bandgap (∼1.5 eV) and significant anisotropic mobility (∼10^3^ cm^2^ V^−1^ s^−1^) but suffers from instability and rapid oxidation in air.^16,17^ TMDs may exhibit strong structural integrity, favorable bandgaps, and excellent on/off ratios; for example, monolayer MoS_2_ has a direct bandgap (∼1.8 eV) and carrier mobility (∼10^2^ cm^2^ V^−1^ s^−1^), yet TMDs still fall short of phosphorene in carrier mobility, hindering their potential applications in FETs.^18–20^ Therefore, there is an imperative need to discover 2D layered optoelectronic materials that harmoniously combine stability, appropriate bandgaps, and high mobility for cutting-edge optoelectronic devices.

Numerous studies have revealed that exploring innovative structures within 2D materials enhances their physical and chemical characteristics, thereby facilitating their widespread application in the fields of optoelectronics and spintronics.^21–27^ For instance, Roy *et al.*^28^ suggested that their attributes and performance can be significantly improved through careful structural and chemical adjustments involving phase, size, composition, defects, dopants, topological, and heterostructure engineering. Wang *et al.*^29^ successfully fabricated of a novel 2D material, M_2_X_3_ (where M = Mo, W; X = S, Se), by introducting structural point/line defects. This material exhibits an appealing band gap of 0.89 eV and impressive electron mobility (∼10^4^ cm^2^ V^−1^ s^−1^) for monolayer MoS_2_, as predicted by first-principles calculations. Additionally, defect engineering indicates that the W_2_Se_3_ monolayer can also achieve ultrahigh and anisotropic electron mobility, reaching as much as 10^4^ cm^2^ V^−1^ s^−1^, significantly surpassing that of monolayer WSe_2_. Recently, a novel 2D material, W_8_Se_12_, was synthesized at elevated temperatures.^29^ This material possesses a layered structure resembling a sandwich, comprising Se–W–Se stacks that closely mimic the crystal arrangement of monolayer WSe_2_. Both Mo_8_S_12_ and Mo_8_Se_12_ monolayers exhibit impressive optical absorption characteristics in the visible spectrum and display high anisotropic in-plane mobility that significantly surpasses that of traditional TMDs.^30^ While these findings establish the potential of the M_8_X_12_ framework, most investigations have focused on binary compounds with fixed stoichiometries. To expand this family, ternary alloying strategies have been proposed, including our recent report on Janus Mo_8_S_6_Se_6_ monolayers.^31^ However, the W-based ternary analog W_8_S_6_Se_6_ has yet to be systematically studied. Building on this foundation, the present work systematically investigates various crystal phases (*P*6*m*2, *P*6*mm*, *P*3*m*1, *Cmm*2, and *C*2/*m*) of the ternary W_8_S_6_Se_6_ monolayer using a sulfur-selenium alloying strategy. It thoroughly explores the effects of elemental alloying on the bandgap and carrier mobility, proposing a novel approach to performance optimization through composition control and crystal phase engineering. This research broadens the design scope of the M_8_X_12_ material system and offers a greater variety of material options for optoelectronic applications.

Carrier mobility is a crucial performance metric for semiconductor materials, as it not only governs charge transport dynamics but also significantly defines the ultimate performance ceiling of nano-optoelectronic devices. Based on this understanding, we conducted a comprehensive investigation of five crystalline phases of W_8_S_6_Se_6_ monolayer materials using first-principles calculations, focusing on their structural stability (encompassing kinetic, thermodynamic, and mechanical stability), elastic characteristics, photoelectric response, and carrier transport properties. Our findings reveal that all crystalline phases demonstrate remarkable stability. Moreover, these materials possess an optimal direct bandgap and a light absorption coefficient exceeding 10^5^ cm^−1^ from the near-infrared to the visible region, alongside an impressive room-temperature electron mobility of up to 10^4^ cm^2^ V^−1^ s^−1^. These exceptional attributes position W_8_S_6_Se_6_ monolayer materials favorably for FETs and high-efficiency photodetectors, highlighting their significant potential for future integration into next-generation optoelectronic devices.

## 2. Calculation methods

The calculations in this work were conducted within the framework of density functional theory (DFT) as implemented in the Vienna *Ab initio* Simulation Package (VASP).^32^ The Perdew–Burke–Ernzerhof (PBE)^33^ generalized gradient approximation was utilized to incorporate exchange-correlation effects. Additionally, to enhance accuracy in band structure analysis, the Heyd–Scuseria–Ernzerhof (HSE06) hybrid functional was applied. A vacuum layer of 20 Å was applied along the perpendicular direction to eliminate interlayer interactions. The plane-wave cutoff energy was established at 500 eV. Geometry optimizations employed a 6 × 6 × 1 grid of *Γ*-centered Monkhorst–Pack *k*-points. These monolayer configurations were completely optimized without applying symmetry restrictions, utilizing the conjugate gradient method. Convergence thresholds were established at 1 × 10^−7^ eV for electronic energy differences and 1 × 10^−4^ eV Å^−1^ for Hellmann–Feynman atomic forces. Single-particle optical calculations were performed using LOPTICS = .T. in VASP. The imaginary dielectric function comes from direct interband transitions under Fermi's golden rule, while intraband transitions are neglected. The real part was obtained *via* Kramers–Kronig transformation, and anisotropic absorption coefficients were derived from the dielectric tensor. For PBE-level single-particle optical calculations, NBANDS = 168 was used to include all interband transitions, with convergence confirmed by band-number tests. For these PBE-level optical dielectric calculations, a denser converged 12 × 12 × 1 *k*-point grid was adopted. CSHIFT = 0.15 eV was applied as a broadening parameter to smooth transition peaks. Local-field corrections were disabled following the default setting of VASP LOPTICS module. For excited-state optical properties, the GW-BSE method was employed, specifically using the G_0_W_0_ approximation for GW. GW parameter convergence tests (*k*-point mesh, ENCUTGW, number of bands, vacuum thickness) were carried out for the representative *P*6*m*2-W_8_S_6_Se_6_ monolayer. BSE calculations reused fully converged GW quasiparticle wavefunctions and eigenvalues, with convergence evaluated based on the lowest singlet excitonic energy. Production BSE calculations adopted a 12 × 12 × 1 *k*-point mesh and NBANDS_BSE_ = 160. These converged parameters were applied to all five polymorphs due to their similar cell dimensions, stoichiometry, and lattice constants. Full convergence criteria and test results are provided in Fig. S1 and S2 of the SI. The carrier mobilities of W_8_S_6_Se_6_ monolayers with different phases are calculatedby the following equation:^34^1
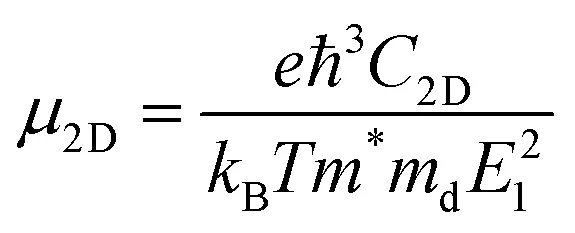
In this context, *C*_2D_ denotes the 2D elastic modulus, derived from *C*_*ij*_, and *T* = 300 K represents the temperature. The effective mass in the direction of transport is represented by m*, while 
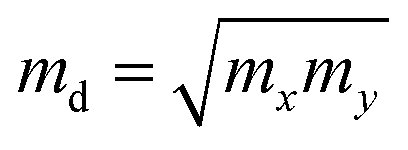
 signifies the average effective mass. Furthermore, the deformation potential constant *E*_i_ is defined as *E*_i_ = Δ*E*/*δ*. Here, Δ*E* denotes the energy shift of the valence band maximum (VBM) or conduction band minimum (CBM) in relation to the vacuum level, and *δ* = Δ*l*/*l* under applied uniaxial strain. We adopt deformation potential theory to calculate mobility, which accounts only for acoustic phonon scattering, excluding optical phonon and impurity scattering. The predicted mobilities represent semi-quantitative upper limits for perfect crystals and are primarily used for comparative analysis between phases.

## 3. Results and discussion

In this work, we identified five stable atomic configurations of monolayer W_8_S_6_Se_6_ with varying spatial group symmetries through a systematic structural search. These 2D structures all display typical hexagonal close-packed (hcp) lattice characteristics, with their primitive basis vectors designated as *a* and *b*, as depicted in Fig. 1. Specifically, the five configurations belong to the following space groups: *P*6*m*2, *P*6*mm*, *P*3*m*1, *Cmm*2, and *C*2/*m*, respectively. All structures exhibit a sandwich-like arrangement with three layers of atoms, in which W atoms occupy the middle layer and S/Se atoms are distributed across the upper and lower surface layers. The optimized geometric parameters of the W_8_S_6_Se_6_ monolayers are detailed in Table 1. Notably, the lattice constants for the five configurations were accurately measured as 9.345 Å, 9.349 Å, 9.341 Å, 9.340 Å, and 9.343 Å, respectively. These values fall within the range of lattice constants for monolayer W_8_S_12_ (9.273 Å) and W_8_Se_12_ (9.556 Å).^30^ To illustrate the anisotropic carrier transport characteristics more clearly, we used olive dashed lines to indicate the different crystalline axis directions (*x* and *y*) in the orthogonal lattice representation.

**Fig. 1:**
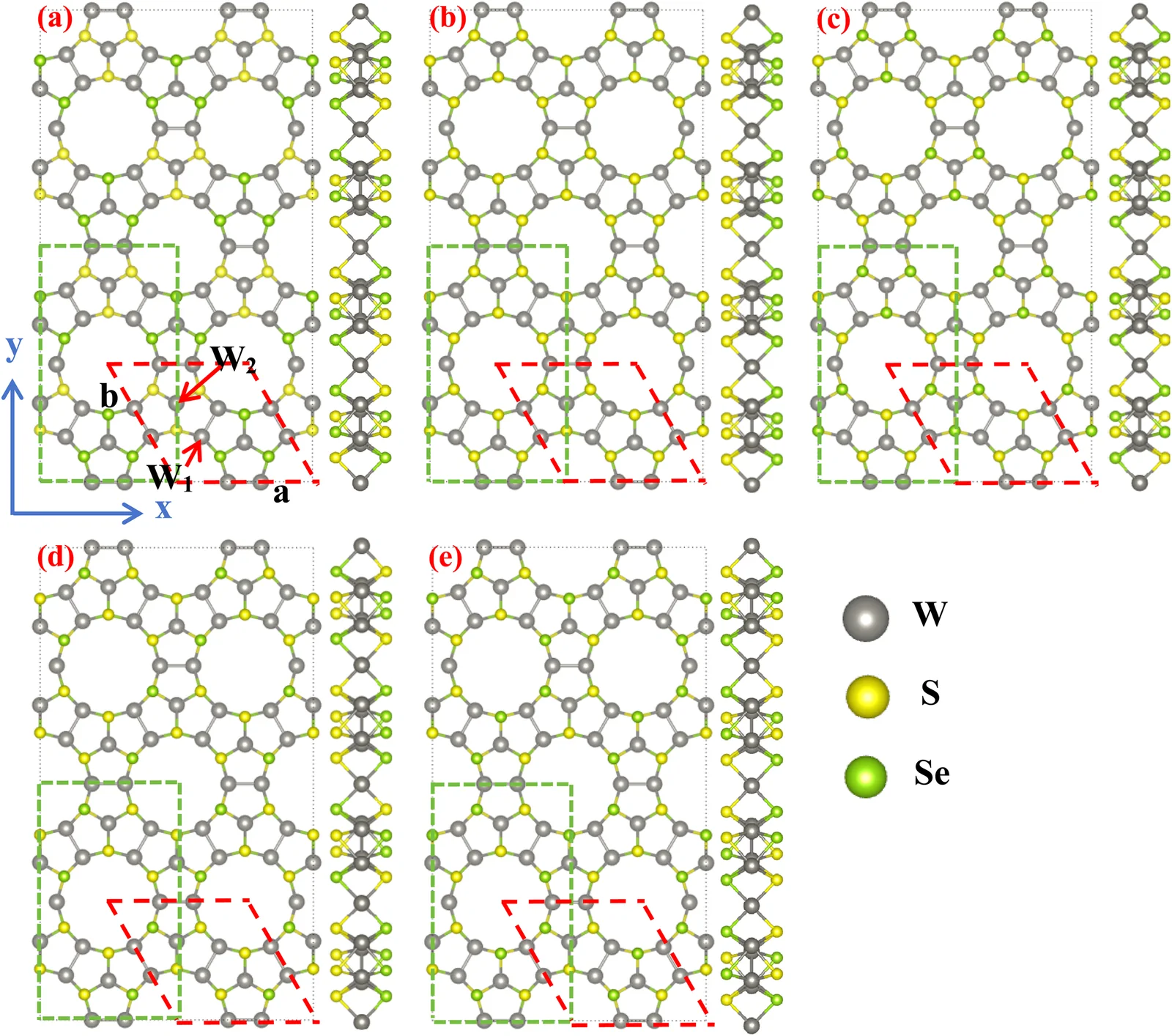
Top and side perspectives of (a) *P*6*m*2, (b) *P*6*mm*, (c) *P*3*m*1, (d) *Cmm*2, and (e) *C*2/*m*-W_8_S_6_Se_6_ monolayers.

**Table 1:** Calculated structural parameters of *P*6*m*2, *P*6*mm*, *P*3*m*1, *Cmm*2, and *C*2/*m*-W_8_S_6_Se_6_ monolayers

W_8_S_6_Se_6_	Lattice constant (Å)	*d*_W1-S_	*d*_W1-Se_	*d*_W2-S_	*d*_W2-Se_	*d*_W1-W1_	*E*_form_ (eV per atom)	*E*_coh_ (eV per atom)	Eg (eV)	
									PBE	HSE
*P*6*m*2	*a* = *b* = 9.345	2.415	2.543	2.470	2.618	2.335	−1.137	−5.799	0.43	1.01
*P*6*mm*	*a* = *b* = 9.349	2.413	2.545	2.487	2.599	2.335	−1.133	−5.795	0.45	1.02
*P*3*m*1	*a* = *b* = 9.341	2.412	2.548	2.467	2.619	2.334	−1.139	−5.802	0.44	1.03
*Cmm*2	*a* = *b* = 9.340	2.413	2.548	2.464	2.625	2.334	−1.137	−5.800	0.43	1.01
*C*2/*m*	*a* = *b* = 9.343	2.409	2.549	2.483	2.603	2.334	−1.136	−5.799	0.43	1.02

To thoroughly evaluate the stability of the various phases of W_8_S_6_Se_6_ monolayers, we initiated our analysis by calculating the cohesive energy for each phase utilizing the equation *E*_coh_ = [*E*_tot_ − (*n*_Mo_*E*_Mo_ + *n*_S_*E*_S_ + *n*_Se_*E*_Se_)]/(*n*_Mo_ + *n*_S_ + *n*_Se_). The computed values are summarized in Table 1. Notably, all configurations exhibit negative cohesive energies, with absolute values surpassing those of WSSe,^35,36^ indicating that the W_8_S_6_Se_6_ monolayers demonstrate remarkable binding stability. To further assess thermodynamic synthesizability and resistance to elemental decomposition, we also calculated the formation energy (*E*_form_) for all five polymorphs, as summarized in Table 1. The *P*6*m*2, *P*6*mm*, *P*3*m*1, *Cmm*2, and *C*2/*m* phases exhibit formation energies of −1.137, −1.133, −1.139, −1.137, and −1.136 eV per atom, respectively. All negative values confirm that these ternary monolayers are thermodynamically stable against decomposition into elemental W, S, and Se, with *P*3*m*1 showing the lowest *E*_form_ and thus the optimal thermodynamic stability. Together, the cohesive energy and formation energy collectively verify the intrinsic energetic stability of the target monolayers. This is further confirmed through phonon spectrum analysis and *ab initio* molecular dynamics (AIMD) simulations. As shown in Fig. 2, no imaginary frequencies are observed throughout the entire Brillouin zone in the phonon dispersion curves, confirming the dynamical stability of the *P*6*m*2, *P*6*mm*, *P*3*m*1, *Cmm*2, and *C*2/*m*-W_8_S_6_Se_6_ monolayers. Additionally, AIMD simulations are regarded as a robust approach for evaluating the materials' thermal stability. Accordingly, a 5 ps AIMD simulation was carried out in the canonical NVT ensemble at room temperature. The atomic configuration of the 4 × 4 × 1 supercell remained intact throughout the simulation, with no bond breakages or significant structural reconstruction observed in the final configurations (see the inset of Fig. S3). These findings unequivocally affirm the outstanding thermodynamic stability of the predicted W_8_S_6_Se_6_ monolayers under ambient conditions.

**Fig. 2:**
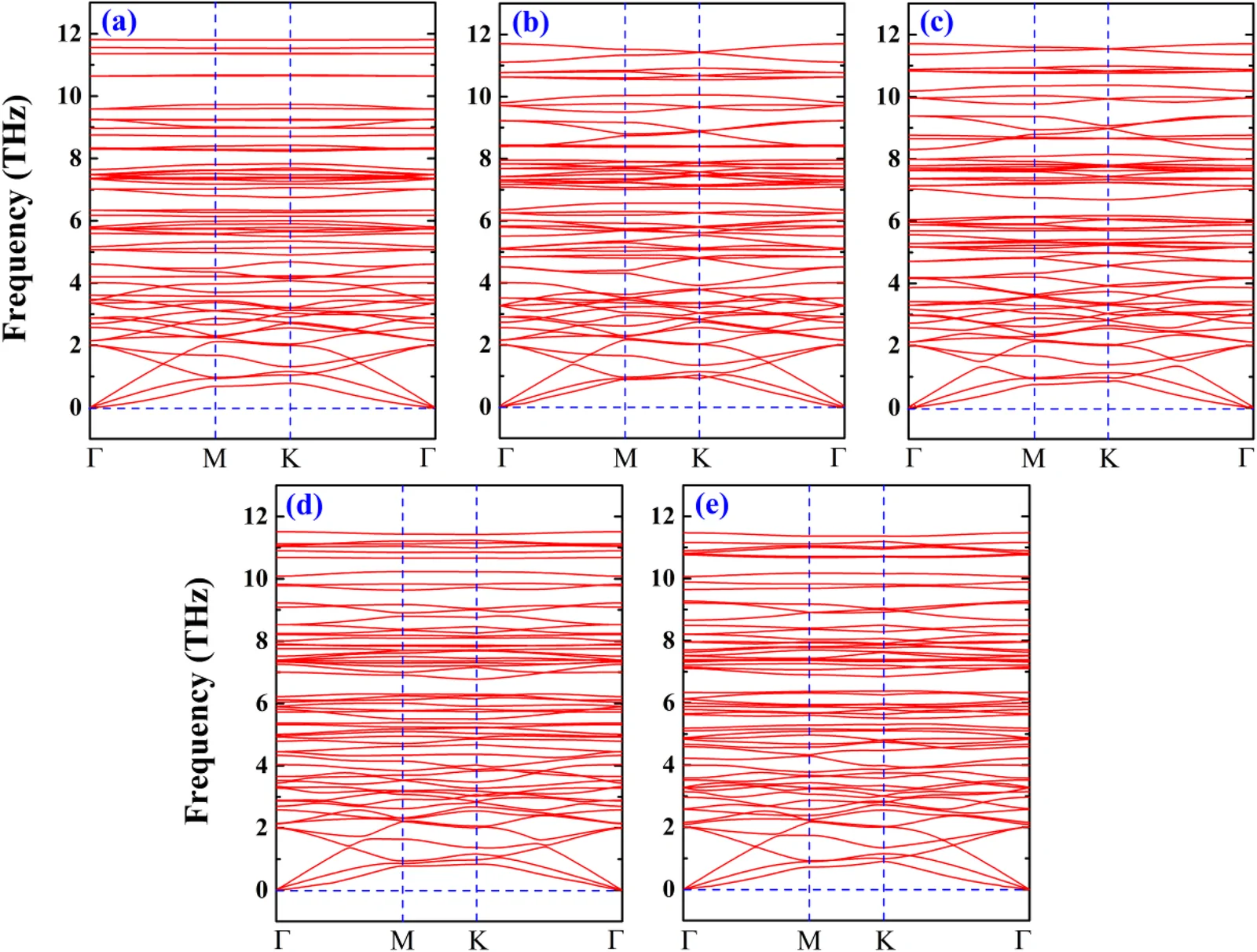
Calculated the phonon dispersion curves of (a) *P*6*m*2, (b) *P*6*mm*, (c) *P*3*m*1, (d) *Cmm*2, and (e) *C*2/*m*-W_8_S_6_Se_6_ monolayers.

To comprehensively evaluate the mechanical characteristics of different crystalline phases in W_8_S_6_Se_6_ monolayers, we commenced by calculating the linear elastic coefficients *C*_*ij*_ through a polynomial fitting approach applied to he energy–strain curves. Given the hexagonal lattice symmetry, only *C*_11_ and *C*_12_ constants were independent, as summarized in Table 2. It is noteworthy that the *C*_*ij*_ values comply with Born's stability criteria relevant to 2D hexagonal systems. Specifically, the conditions *C*_11_ > 0 and *C*_66_ = (*C*_11_ − *C*_12_)/2 > 0 (ref. 37 and 38) are unequivocally met, thereby confirming the mechanical stability of the *P*6*m*2, *P*6*mm*, *P*3*m*1, *Cmm*2, and *C*2/*m*-W_8_S_6_Se_6_ monolayers. Furthermore, recognizing the importance of Poisson's ratio *ν* and in-plane Young's modulus *Y*_s_ for predicting mechanical behavior under practical loading conditions, we focused on determining these essential parameters. They were derived from the obtained elastic constants using the following relations:^39,40^
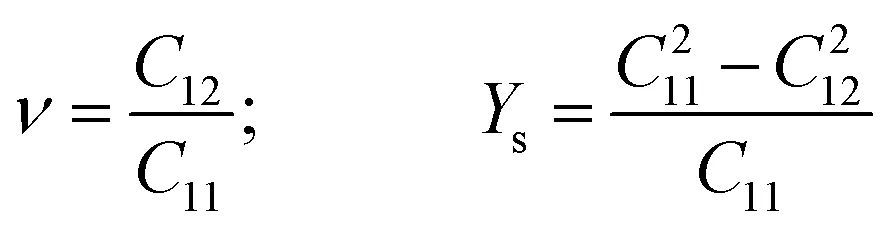
Table 2 shows the computed *Y*_s_ values for the *P*6*m*2, *P*6*mm*, *P*3*m*1, *Cmm*2, and *C*2/*m*-W_8_S_6_Se_6_ monolayers, which are 55.8 N m^−1^, 55.5 N m^−1^, 55.63 N m^−1^, 55.39 N m^−1^, and 55.39 N m^−1^, respectively. Each of these values is noticeably lower than the *Y*_s_ value of the WSSe monolayer.^41^ These findings imply that the W_8_S_6_Se_6_ monolayers are more flexible compared to the Janus monolayer WSSe, and the reduced *Y*_s_ further supports the conclusion that these structures possess significant mechanical pliability. Moreover, the computed *ν* values for the *P*6*m*2, *P*6*mm*, *P*3*m*1, *Cmm*2, and *C*2/*m*-W_8_S_6_Se_6_ monolayers are 0.512, 0.435, 0.431, 0.433, and 0.434, respectively, suggesting that all these W_8_S_6_Se_6_ monolayers exhibit ductile behavior. These computational outcomes provide a significant theoretical foundation for employing W_8_S_6_Se_6_ monolayers in ductile electronic devices.

**Table 2:** Calculated elastic parameters (*C*_*ij*_, *Y*_s_ and *ν*) of *P*6*m*2, *P*6*mm*, *P*3*m*1, *Cmm*2, and *C*2/*m*-W_8_S_6_Se_6_ monolayers

W_8_S_6_Se_6_	*C*_11_ (N m^−1^)	*C*_12_ (N m^−1^)	*C*_66_ (N m^−1^)	*Y*_s_ (N m^−1^)	*ν*
*P*6*m*2	75.58	38.67	18.46	55.80	0.512
*P*6*mm*	68.47	29.80	19.34	55.50	0.435
*P*3*m*1	68.30	29.41	19.44	55.63	0.431
*Cmm*2	68.17	29.52	19.33	55.39	0.433
*C*2/*m*	68.25	29.62	19.31	55.39	0.434

2D materials exhibiting direct bandgaps are vital for optoelectronic applications due to their superior light absorption efficiency and capabilities for radiative recombination. To explore this further, we undertook an electronic structure analysis of the proposed *P*6*m*2, *P*6*mm*, *P*3*m*1, *Cmm*2, and *C*2/*m*-W_8_S_6_Se_6_ monolayers, utilizing various computational functionals, as depicted in Fig. 3. Our calculations employing the PBE functional revealed that all five monolayer configurations possess direct bandgaps, ranging from 0.43 eV to 0.45 eV. However, acknowledging the PBE functional's known tendency to underestimate bandgaps in 2D semiconductors, we adopted the more precise HSE06 hybrid functional to obtain reliable electronic properties. Taking the *P*6*m*2-W_8_S_6_Se_6_ monolayer as a representative case (Fig. 3a), we observed that the band structure determined *via* HSE06 closely resembles that obtained through the PBE method, yet the bandgap increases to 1.01 eV. Consequently, we re-evaluated the electronic structures of all W_8_S_6_Se_6_ monolayers utilizing the HSE06 method, with the corresponding bandgap values compiled in Table 1. The findings indicate that the W_8_S_6_Se_6_ monolayers in the *P*6*mm*, *P*3*m*1, *Cmm*2, and *C*2/*m* configurations also behave as direct bandgap semiconductors, exhibiting bandgaps of 1.02 eV, 1.03 eV, 1.01 eV, and 1.01 eV at the HSE06 level, respectively (Fig. 3b–e). Notably, both the VBM and CBM for these monolayers are located at the *Γ* point. To gain a more profound insight into their electronic structure, we analyzed the orbital contributions to the VBM and CBM, with a particular focus on the *P*6*m*2 phase. From the partial density of states (PDOS) (Fig. 4a) and the projected band structure (Fig. S4a), we observed that the CBM of the *P*6*m*2-W_8_S_6_Se_6_ monolayer primarily originates from the d_z^2^_ orbitals of W atoms, while the VBM is chiefly comprised of the degenerate d_xz_/d_yz_ orbitals of W atoms. As shown in Fig. 4b–e and S4b–e, the orbital contributions of the VBM and CBM in the other configurations align with those observed in the *P*6*m*2 phase. We further evaluated spin–orbit coupling (SOC) effects from the heavy W and Se atoms using PBE + SOC calculations for all phases (Fig. S5, Table SI). SOC slightly widens the band gap by approximately 10–20 meV, without shifting the *Γ*-located band edges or altering VBM/CBM orbital characters. To further clarify the bonding features and charge redistribution beyond band structure and PDOS, we calculated the charge density difference for the five polymorphic W_8_S_6_Se_6_ monolayers (Fig. 5). The central W atoms exhibit obvious charge depletion (olive isosurface), while the surrounding S and Se chalcogen sites show prominent charge accumulation (red isosurface), confirming electron transfer from W to the chalcogen atoms driven by electronegativity discrepancy. Connected charge density contours bridging W and S/Se atoms demonstrate strong hybridization between W d-orbitals and S/Se p-orbitals, which facilitates the formation of robust polar covalent bonds. The asymmetric S/Se distribution on the two surfaces creates a vertical charge imbalance, an out-of-plane dipole, and a built-in electric field, accounting for anisotropic optical behaviors. Crystal symmetry breaking tunes the charge distribution: the high-symmetry *P*6*mm* phase features homogeneous charge transfer, whereas the *P*6*m*2, *P*3*m*1, *Cmm*2, and *C*2/*m* present prominent in-plane charge anisotropy. Differences in orbital hybridization modulate the band gap, carrier effective mass, and optical absorption of each phase. Additionally, we initially calculated anisotropic absorption coefficients *via* the single-particle PBE approximation, as shown in Fig. S6. However, PBE neglects electron–hole excitonic coupling and underestimates polarization anisotropy, thus failing to reproduce the intrinsic optical response of 2D semiconductors. To address this, we performed GW-BSE calculations to account for excitonic effects, with the GW-BSE absorption spectra presented in Fig. 6. The GW-BSE results reveal that these monolayers possess absorption coefficients up to 10^5^ cm^−1^ across the near-infrared to visible spectrum, comparable to those of WS_2_ and WSSe,^42,43^ thereby underscoring their potential applications in solar cells and infrared photodetectors. The GW-BSE spectra feature distinct excitonic resonant peaks and red-shifted absorption edges. Combined with PDOS and charge density difference analyses, the stronger light absorption along the *y*-axis originates from highly delocalized W d_z^2^_ orbitals, which is consistent with the superior *y*-direction electron mobility of all five polymorphs. Variations in crystal symmetry and the asymmetric S/Se distribution induce distinguishable excitonic spectral characteristics among the five W_8_S_6_Se_6_ phases.

**Fig. 3:**
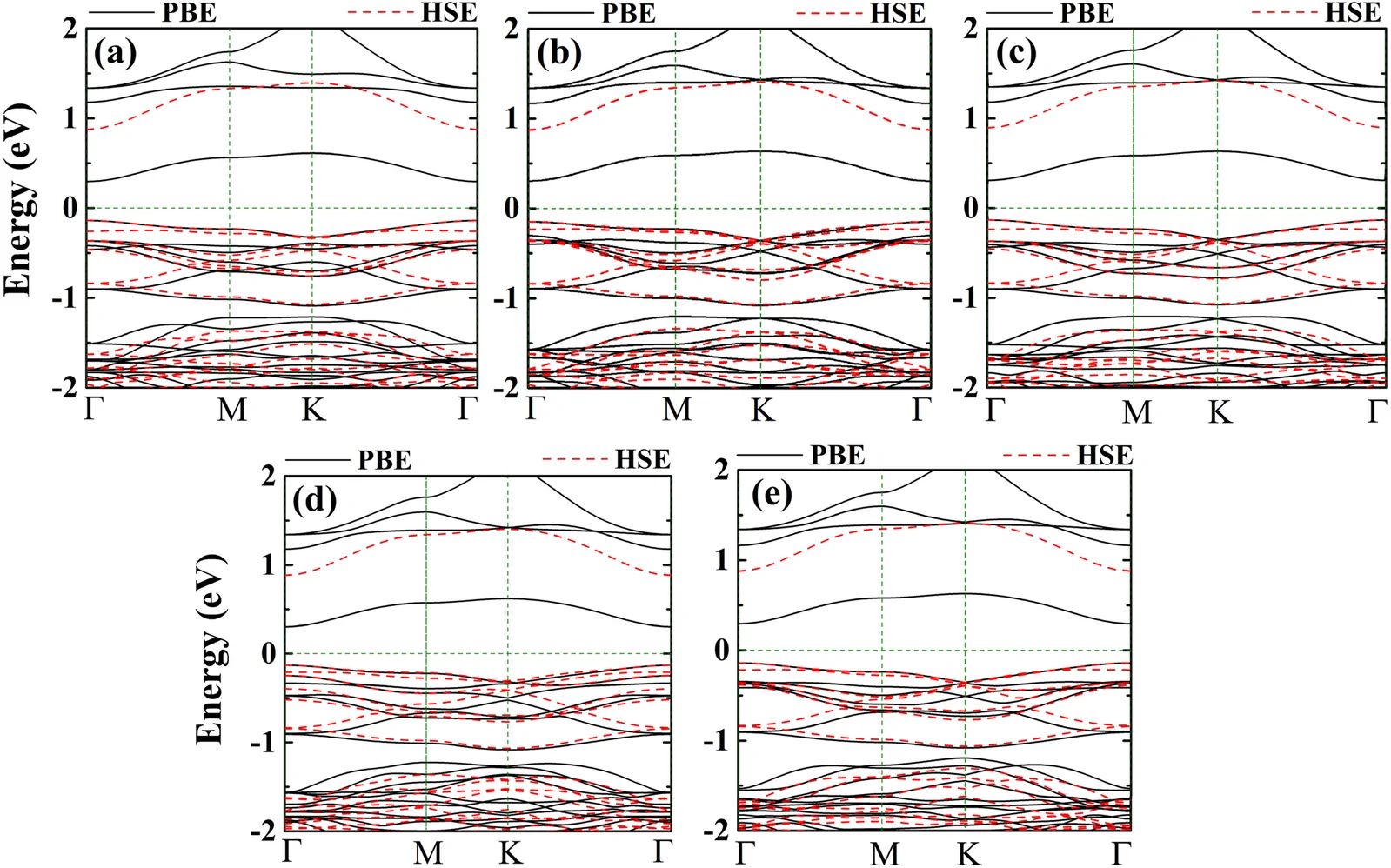
Band structures of (a) *P*6*m*2, (b) *P*6*mm*, (c) *P*3*m*1, (d) *Cmm*2, and (e) *C*2/*m*-W_8_S_6_Se_6_ monolayers by utilizing the PBE (black) and HSE06 (red) functionals.

**Fig. 4:**
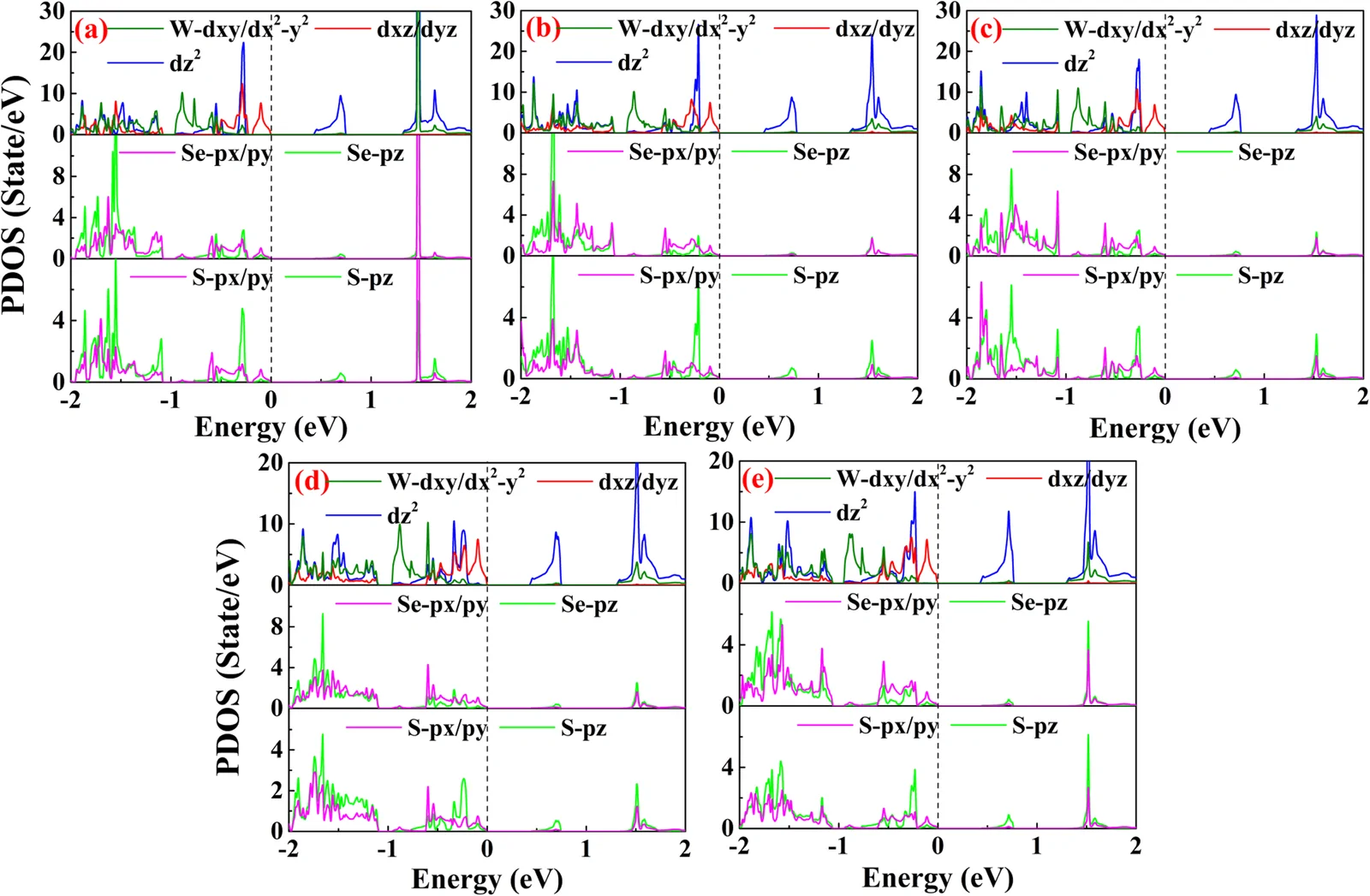
PDOS of (a) *P*6*m*2, (b) *P*6*mm*, (c) *P*3*m*1, (d) *Cmm*2, and (e) *C*2/*m*-W_8_S_6_Se_6_ monolayers.

**Fig. 5:**
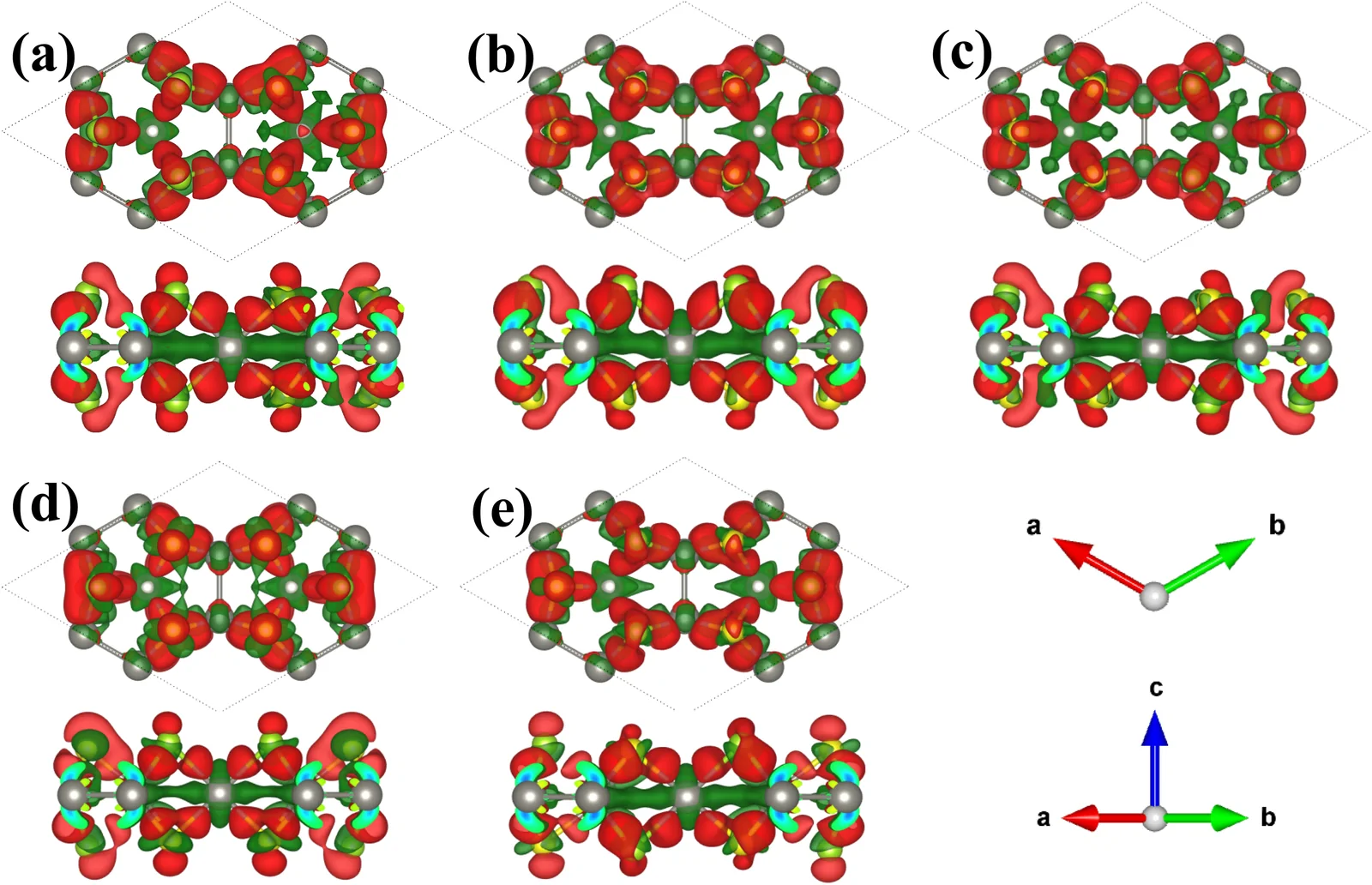
Differential charge density distribution of (a) *P*6*m*2, (b) *P*6*mm*, (c) *P*3*m*1, (d) *Cmm*2, and (e) *C*2/*m*-W_8_S_6_Se_6_ monolayers (isosurface = 0.006 e/Å^3^). The upper panels are top views along the *c*-axis (*ab*-plane), and the lower panels are side views along the in-plane direction. Red isosurfaces represent charge accumulation, olive isosurfaces represent charge depletion.

**Fig. 6:**
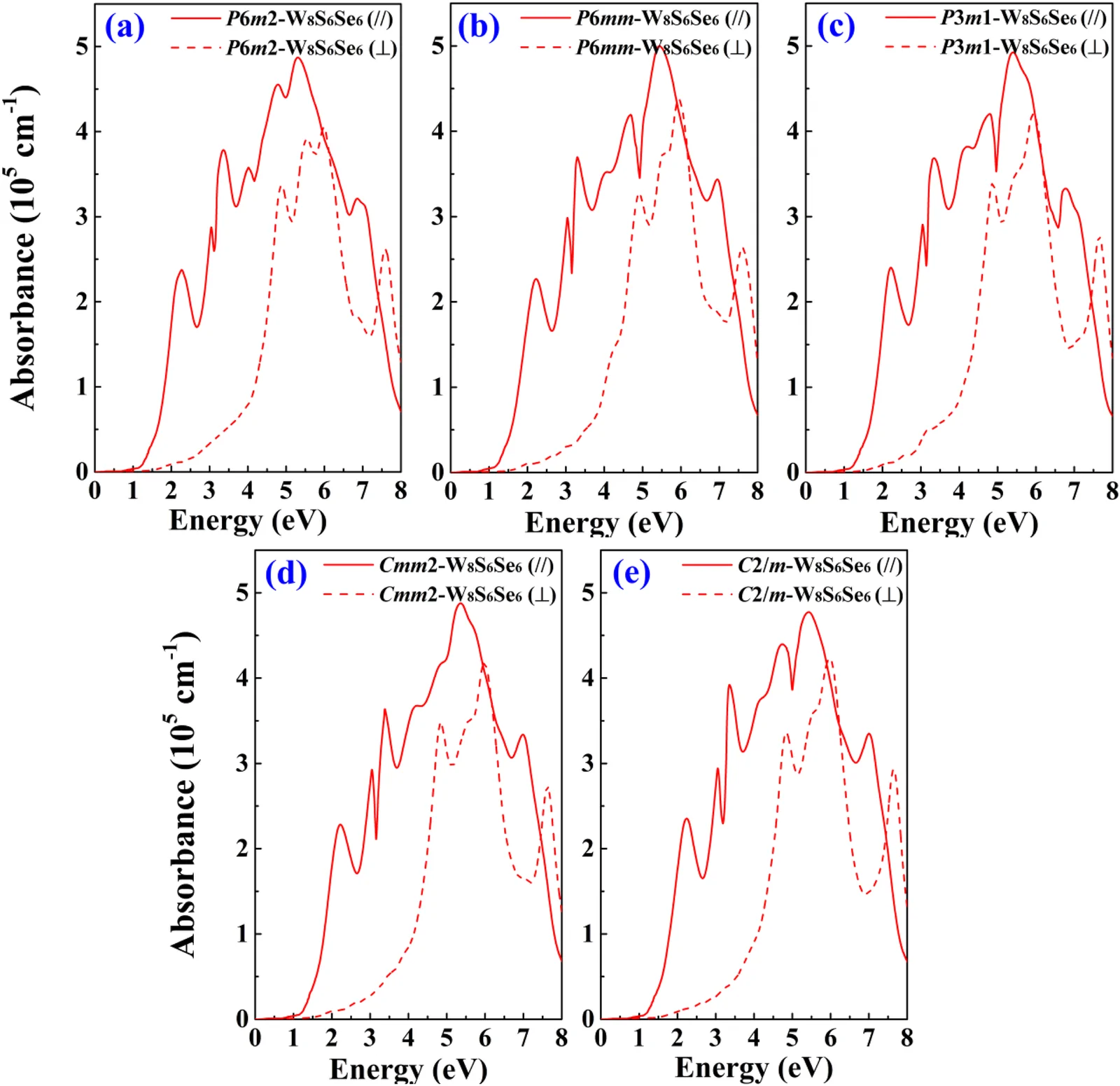
GW-BSE calculated anisotropic absorption coefficients for in-plane (//) and out-of-plane (⊥) polarizations of *P*6*m*2, *P*6*mm*, *P*3*m*1, *Cmm*2, and *C*2/*m*-W_8_S_6_Se_6_ monolayers.

Optimizing carrier transport efficiency is essential for their application in advanced microelectronic devices for 2D semiconductor materials. This characteristic enables that photogenerated carriers (holes and electrons) quickly engage in target physical processes following light absorption, thereby significantly improving photoelectric conversion efficiency. According to deformation potential theory, carrier mobility is predominantly influenced by three critical factors: m*, *E*_i_, and *C*_2D_. As depicted in Fig. 1, the axes of the orthogonal coordinate systems are labeled as the *x*-axis and *y*-axis, respectively, for the five monolayers structures: *P*6*m*2, *P*6*mm*, *P*3*m*1, *Cmm*2, and *C*2/*m*-W_8_S_6_Se_6_. By analyzing the energy–strain relationship, we derived the elastic moduli for these monolayer structures, and the results are presented in Fig. S7 and S8. Furthermore, through a linear regression analysis of the energy fluctuations of the VBM and CBM under applied strain, we determined the deformation potential constants for these five configurations, as illustrated in Fig. S9 and S10. The computed transport parameters (m*, *E*_i_, and *C*_2D_), along with the carrier mobilities in both directions, are summarized in Table 3. A key finding across all five configurations is the pronounced anisotropy in carrier transport. Electron mobility along the *y*-direction consistently exceeds that along the *x*-direction, reaching remarkable values of 2.44 × 10^4^, 3.15 × 10^4^, 2.37 × 10^4^, 2.04 × 10^4^, and 2.06 × 10^4^ cm^2^V^−1^s^−1^ for the *P*6*m*2, *P*6*mm*, *P*3*m*1, *Cmm*2, and *C*2/*m*-W_8_S_6_Se_6_ monolayers, respectively. To assess the effect of SOC, we recalculated the carrier effective mass, deformation potential, 2D elastic constant and mobility within the PBE + SOC framework (Table 3). Spin–orbit coupling causes only minor numerical fluctuations in the transport parameters, and the prominent anisotropy for electrons and holes is completely retained. This superior performance is primarily attributed to the exceptionally low effective mass of electrons in the *y*-direction, a characteristic shared by all five crystal phases. To fully clarify the underlying physical mechanism, we further analyze the correlation between band orbital features, band dispersion, effective mass, and deformation potential below. As shown in Fig. 4 and S4, the CBM of all W_8_S_6_Se_6_ monolayers is dominated by highly delocalized W d_z^2^_ orbitals, while the VBM mainly consists of W d_xz_/d_yz_ orbitals. Stronger interatomic overlap of W d_z^2^_ along the *y*-axis generates steeper band dispersion in Fig. S11, whereas weak *x*-axis orbital coupling flattens bands. The less directional overlap of VBM orbitals also explains the larger effective mass and lower mobility of holes. Band curvature directly determines the carrier effective mass. According to Table 3, *y*-direction electron effective masses (0.093–0.101 *m*_0_) are significantly smaller than those in the *x*-direction (0.287–0.306 *m*_0_). These ultra-light electrons, originating from steep bands along the *y*-axis, are the primary contributors to the outstanding transport performance. Based on deformation potential theory, *E*_i_ fitted *via* strain-dependent band-edge shifts (Fig. S9, S10) are slightly larger along *y*-direction, which slightly enhances electron-phonon scattering. However, mobility is quadratically inversely proportional to m*, so the sharp reduction in *m*_y_^*^ fully compensates for this adverse effect. The 2D elastic modulus extracted from Fig. S7 and S8 shows only weak *x*/*y* anisotropy and barely affects carrier mobility, with minor variations in elastic and deformation parameters across different phases exerting a negligible overall influence on transport. This orbital-driven band anisotropy ultimately results in ultrahigh electron mobility along the *y*-direction. These electron mobility values significantly surpass those of WSSe monolayers and are on par with the capabilities of β-Mo_8_S_6_Se_6_ monolayers.^31,44–46^ Consequently, these W_8_S_6_Se_6_ monolayers, characterized by their improved carrier mobility, present exciting opportunities for the development of high-frequency and low-power FETs. It should be noted that the mobilities reported here are obtained within the DP theory framework, which considers only acoustic phonon scattering. The exclusion of optical phonon scattering and extrinsic scattering sources yields ideal upper-bound mobility values for defect-free samples. Nevertheless, these data provide a reliable basis for comparing the relative anisotropic transport behaviors of the five W_8_S_6_Se_6_ polymorphs, which is the primary objective of this theoretical study.

**Table 3:** Calculated carrier mobilities *µ*_i_ and related parameters (*m*_i_*, *E*_i_, and *C*_i_)of the *P*6*m*2, *P*6*mm*, *P*3*m*1, *Cmm*2, and *C*2/*m*-W_8_S_6_Se_6_ monolayers

Phase	Carrier type	*m*_i_*/*m*_0_		*E*_i_ (eV)		*C*_i_ (N m^−1^)		*µ*_i_ (cm^2^ V^−1^ s^−1^) 10^4^	
		*m*_x_*	*m*_y_*	*E*_*x*_	*E*_*y*_	*C*_*x*_	*C*_*y*_	*µ*_*x*_	*µ*_*y*_
*P*6*m*2-W_8_S_6_Se_6_	Electron	0.306	0.101	1.52	1.54	47.87	48.06	0.82	2.44
	Hole	0.672	0.221	0.89	0.90	47.87	48.06	0.49	1.49
	Electron	0.287	0.094	1.49	1.52	48.21	48.52	0.97	2.89
	Hole	0.792	0.261	1.06	1.08	48.21	48.52	0.25	0.75
*P*6*mm*-W_8_S_6_Se_6_	Electron	0.291	0.096	1.34	1.42	48.32	47.97	1.18	3.15
	Hole	0.685	0.225	0.65	0.70	48.32	47.97	0.91	2.36
	Electron	0.276	0.094	1.26	1.34	48.65	48.29	1.47	3.82
	Hole	0.815	0.260	0.80	0.94	48.65	48.29	0.43	0.97
*P*3*m*1-W_8_S_6_Se_6_	Electron	0.297	0.098	1.51	1.61	48.22	47.99	0.89	2.37
	Hole	0.662	0.218	0.99	1.06	48.22	47.99	0.41	1.10
	Electron	0.266	0.088	1.44	1.56	48.53	48.39	1.23	3.16
	Hole	0.917	0.304	1.19	1.27	48.53	48.39	0.15	0.39
*Cmm*2-W_8_S_6_Se_6_	Electron	0.303	0.099	1.33	1.70	47.87	47.70	1.09	2.04
	Hole	0.707	0.217	0.69	1.11	47.87	47.70	0.76	0.97
	Electron	0.284	0.096	1.25	1.60	48.14	47.99	1.39	2.53
	Hole	0.933	0.416	0.83	1.33	48.14	47.99	0.25	0.22
*C*2/*m*-W_8_S_6_Se_6_	Electron	0.287	0.093	1.60	1.58	47.26	46.70	0.84	2.60
	Hole	0.657	0.219	0.90	0.94	47.26	46.70	0.49	1.37
	Electron	0.273	0.089	1.56	1.49	47.53	46.95	0.98	3.25
	Hole	0.846	0.284	1.09	1.12	47.53	46.95	0.21	0.57

## 4. Conclusion

In summary, we have employed first-principles calculations to predict and analyze the essential characteristics of the *P*6*m*2, *P*6*mm*, *P*3*m*1, *Cmm*2, and *C*2/*m*-W_8_S_6_Se_6_ monolayers. Our investigations, which include assessments of cohesive energy, phonon dispersion, AIMD simulations, and elastic coefficients, reveal that these monolayers exhibit remarkable stability. Furthermore, the computed band gaps for the *P*6*m*2, *P*6*mm*, *P*3*m*1, *Cmm*2, and *C*2/*m*-W_8_S_6_Se_6_ monolayers are 1.01 eV, 1.02 eV, 1.03 eV, 1.01 eV, and 1.01 eV, respectively, as obtained from HSE06 functional calculations. Additionally, the PDOS and projected band structures reveal that VBM for all W_8_S_6_Se_6_ monolayers is predominantly influenced by the d_xz_/d_yz_ orbitals of W atoms, while the CBM predominantly stems from the d_z^2^_ orbital of the W atoms. These monolayer structures also demonstrate ductility and outstanding light absorption coefficients (10^5^ cm^−1^) across the near-infrared to visible spectrum, as demonstrated by GW-BSE calculations. Notably, owing to their low effective mass of electrons in the *y*-direction, all W_8_S_6_Se_6_ monolayers display significant anisotropic in-plane electron mobility, attaining values as high as 10^4^ cm^2^ V^−1^ s^−1^, surpassing those observed in the *x*-direction. Furthermore, the findings of this research highlight the potential of these W_8_S_6_Se_6_ monolayers for innovative applications in advanced flexible optoelectronics and FETs.

## Conflicts of interest

There are no conflicts to declare.

## Supplementary Material

RA-016-D6RA03466G-s001

## Data Availability

All data supporting the findings of this study are available within the paper and its supplementary information (SI). Supplementary information is available. See DOI: https://doi.org/10.1039/d6ra03466g.
