# Supplementary material for: Two-dimensional W8S6Se6 monolayers: intrinsic semiconductors with high anisotropic carrier mobility and optoelectronic performance from first-principles
Source: RSC Adv. 2026 Sep 21;16(44):56061–70. doi: 10.1039/d6ra03466g (PMC13592196; doi:10.1039/d6ra03466g)
Supplement: Supplementary file 1 [file RA-016-D6RA03466G-s001.pdf]

Supplementary Information (SI) for RSC Advances.
This journal is © The Royal Society of Chemistry 2026

Two-Dimensional $W_8S_6Se_6$ Monolayers: Intrinsic Semiconductors with High Anisotropic Carrier Mobility and Optoelectronic Performance from First-Principles

Yi Peng [a] ; Minjia Yao[a]; Fangyuan Li [a]; Yu Chen [a]; Yana Li [a]; Shuangmei Qian [a] Qianqian Zhu*[a]; Juexian Cao*[b]

a: *Microelectronics and Optoelectronics Technology Key Laboratory of Hunan Higher Education, School of Physics and Electronic Electrical Engineering, Xiangnan University, Chenzhou 423000, P. R. China*

b: *Hunan Institute of Advanced Sensing and Information Technology, Xiangtan University, Xiangtan 411105, PR China*

* E-mail: pengzhu2022@ yeah.net

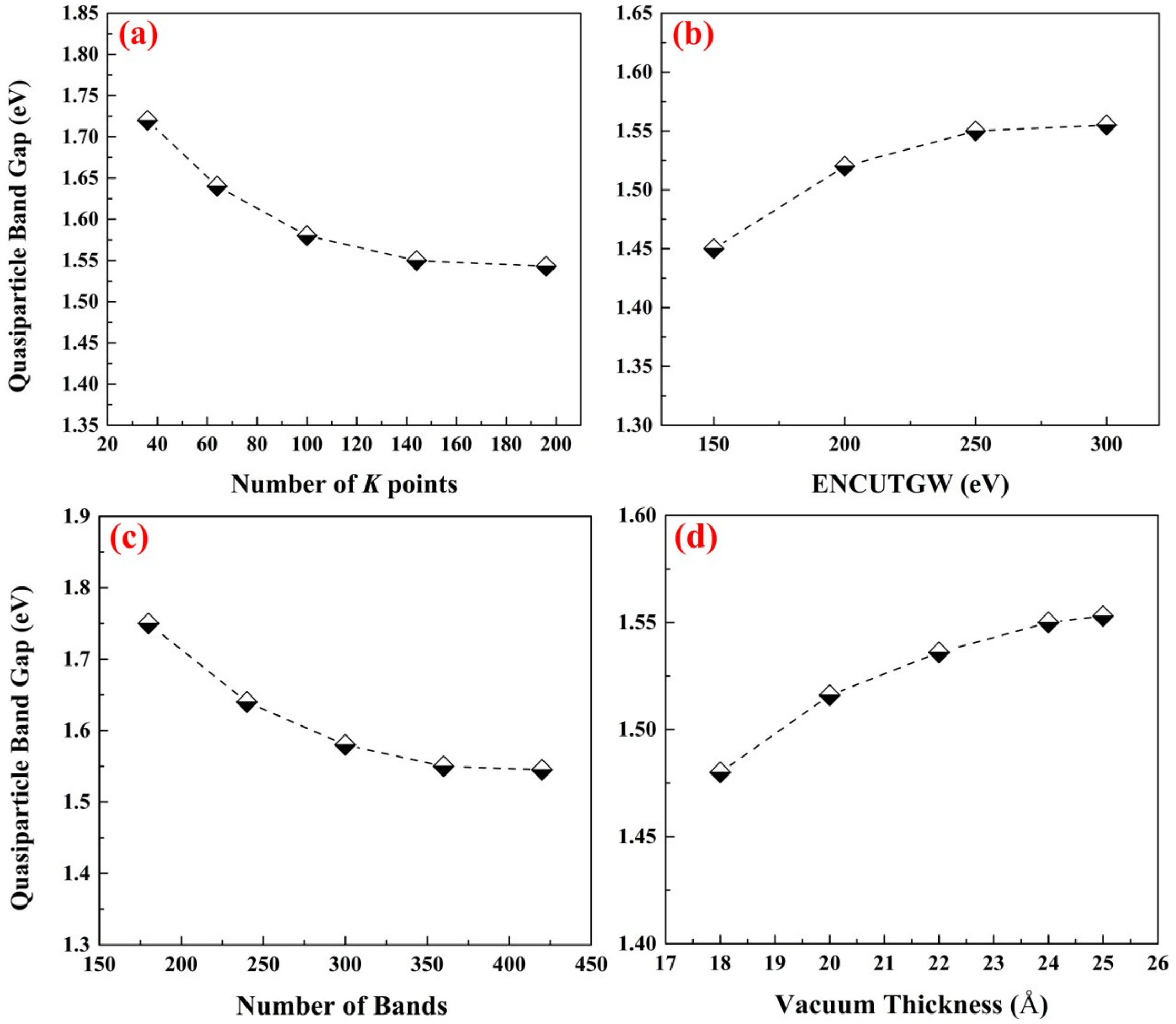

Fig. S1. Convergence tests of the quasiparticle band gap of *P6m2*-$W_8S_6Se_6$ monolayer with respect to (a) k-point numbers, (b) GW cutoff energy, (c) the number of bands, and (d) the vacuum thickness (A parameter is considered converged when further increasing its value induces quasiparticle band-gap variations below 25 meV. The converged GW settings are adopted for GW-BSE calculations of all five$W_8S_6Se_6$ monolayers).

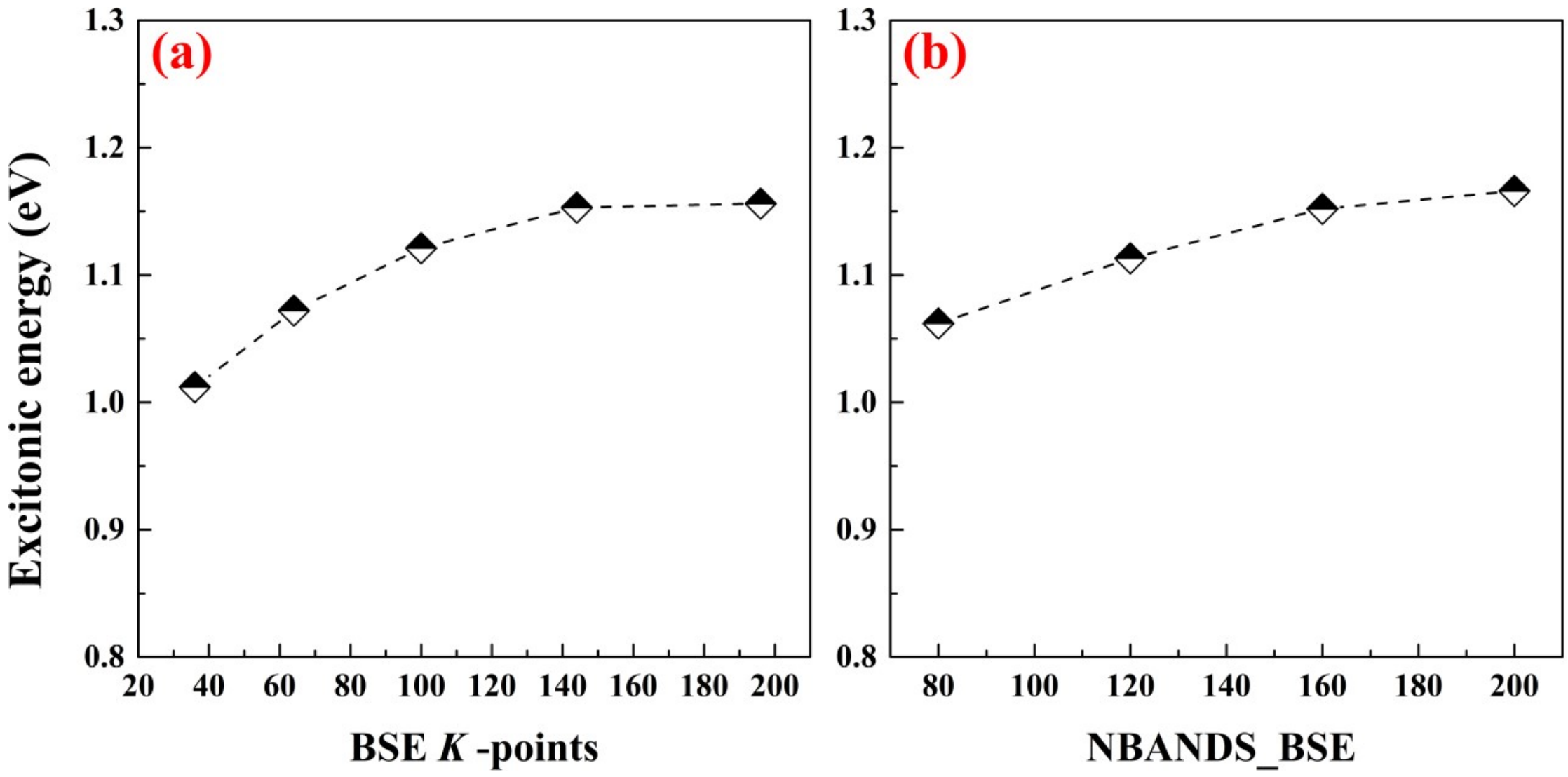

Fig. S2. Convergence of excitonic energy for *P6m2*-$W_8S_6Se_6$ monolayer from BSE calculations. (a) Dependence on total Monkhorst-Pack k-points; (b) Dependence on NBANDS_BSE (All BSE calculations use fixed converged GW quasiparticle wavefunctions without rerunning GW. A convergence criterion of 50 meV is adopted. Final BSE calculations are performed with 12×12×1 k-mesh and NBANDS_BSE = 160).

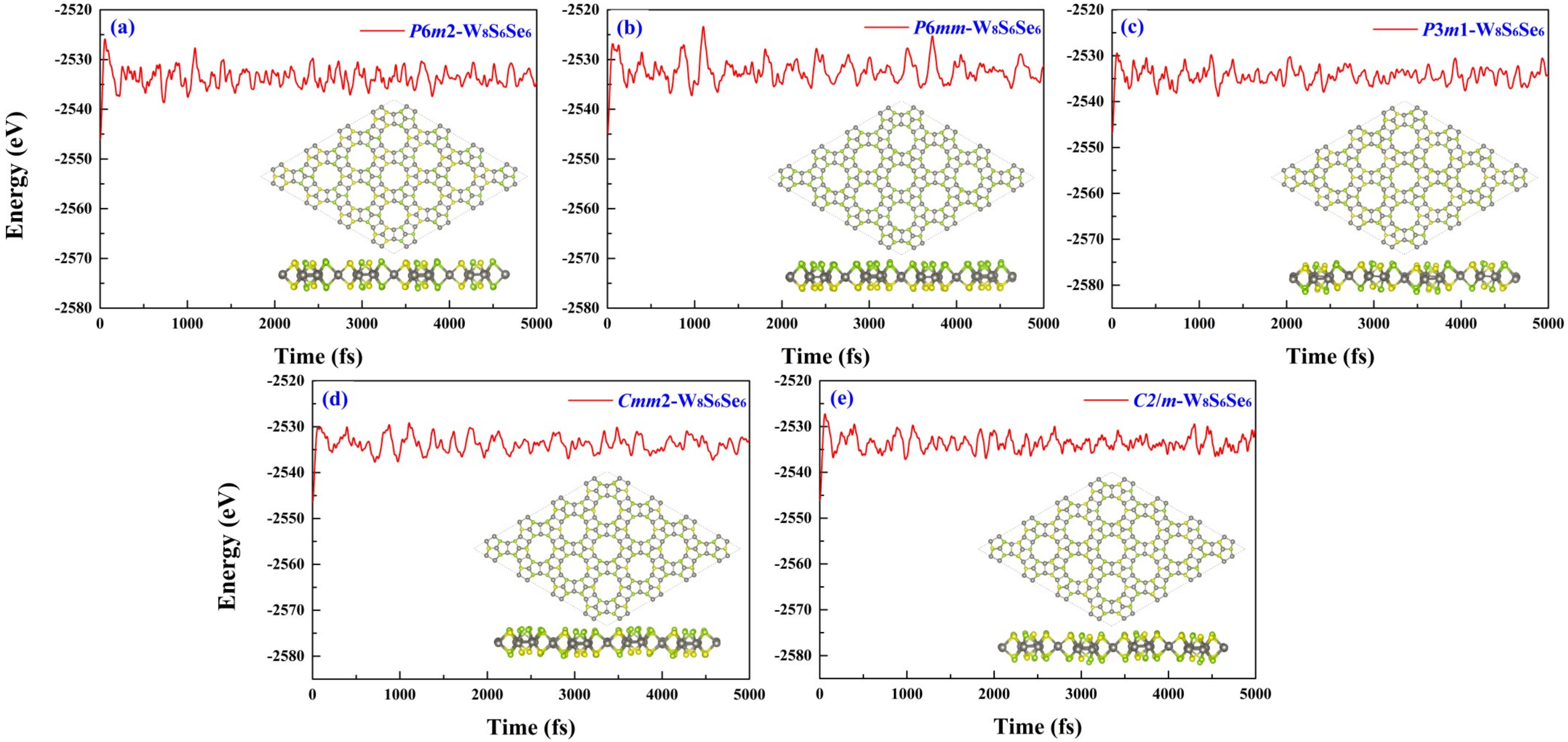

Fig. S3. Calculated AIMD simulations for (a) *P6m2*, (b) *P6mm*, (c) *P3m1*, (d) *Cmm2, and* (e) *C2/m*-$W_8S_6Se_6$ monolayers.

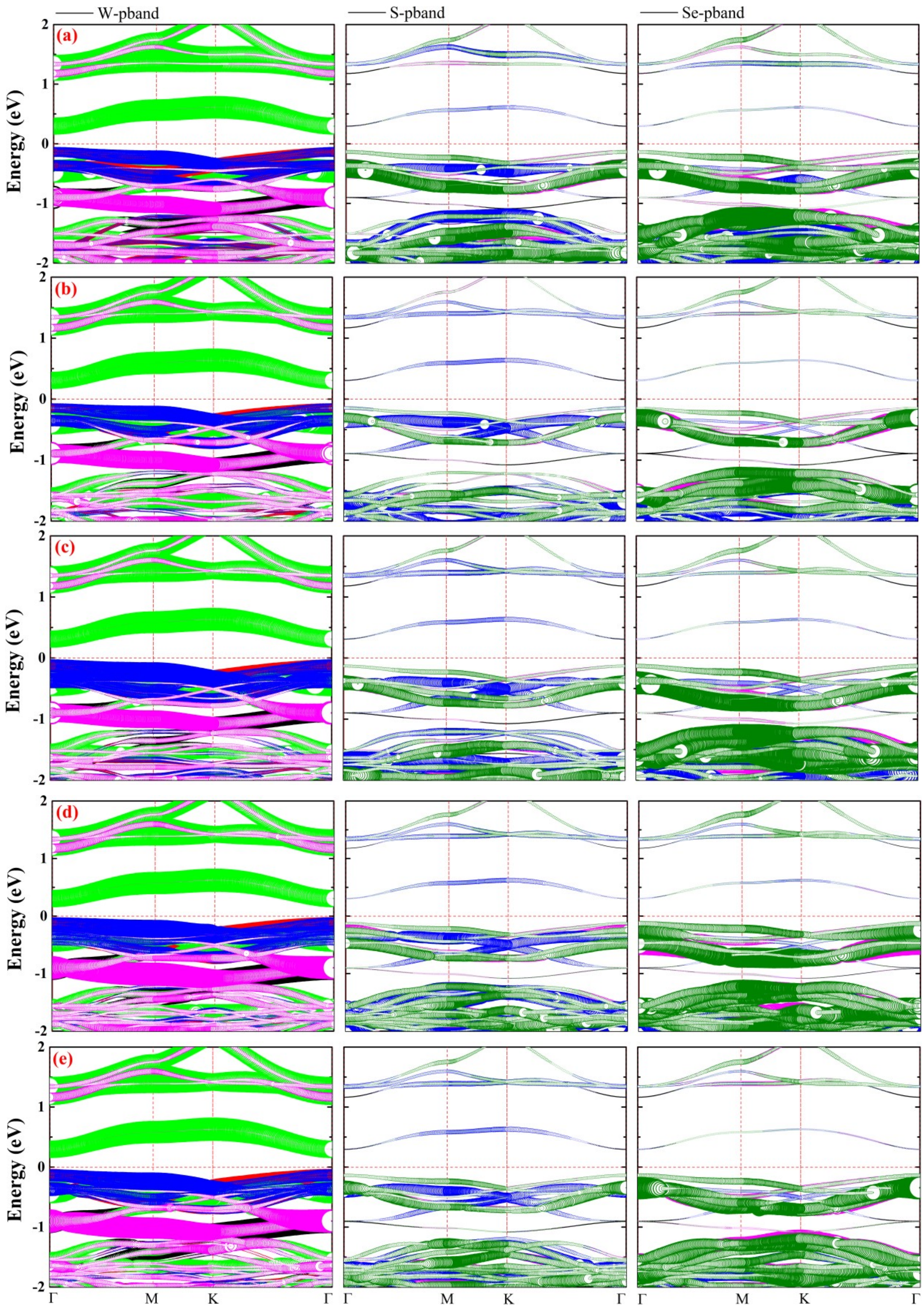

Fig. S4. The orbital-projected band structures of (a) *P6m2*, (b) *P6mm*, (c) *P3m1*, (d) *Cmm2, and* (e) *C2/m*-$W_8S_6Se_6$ monolayers. (The black, red, green, blue, and magenta colors represent the dxy, dyz, dz^2, dxz, and dx^2-y^2 orbitals of the W atom, respectively, while the magenta, blue, and olive colors denote the py, pz, and px orbitals of the S and Se atoms, respectively.)

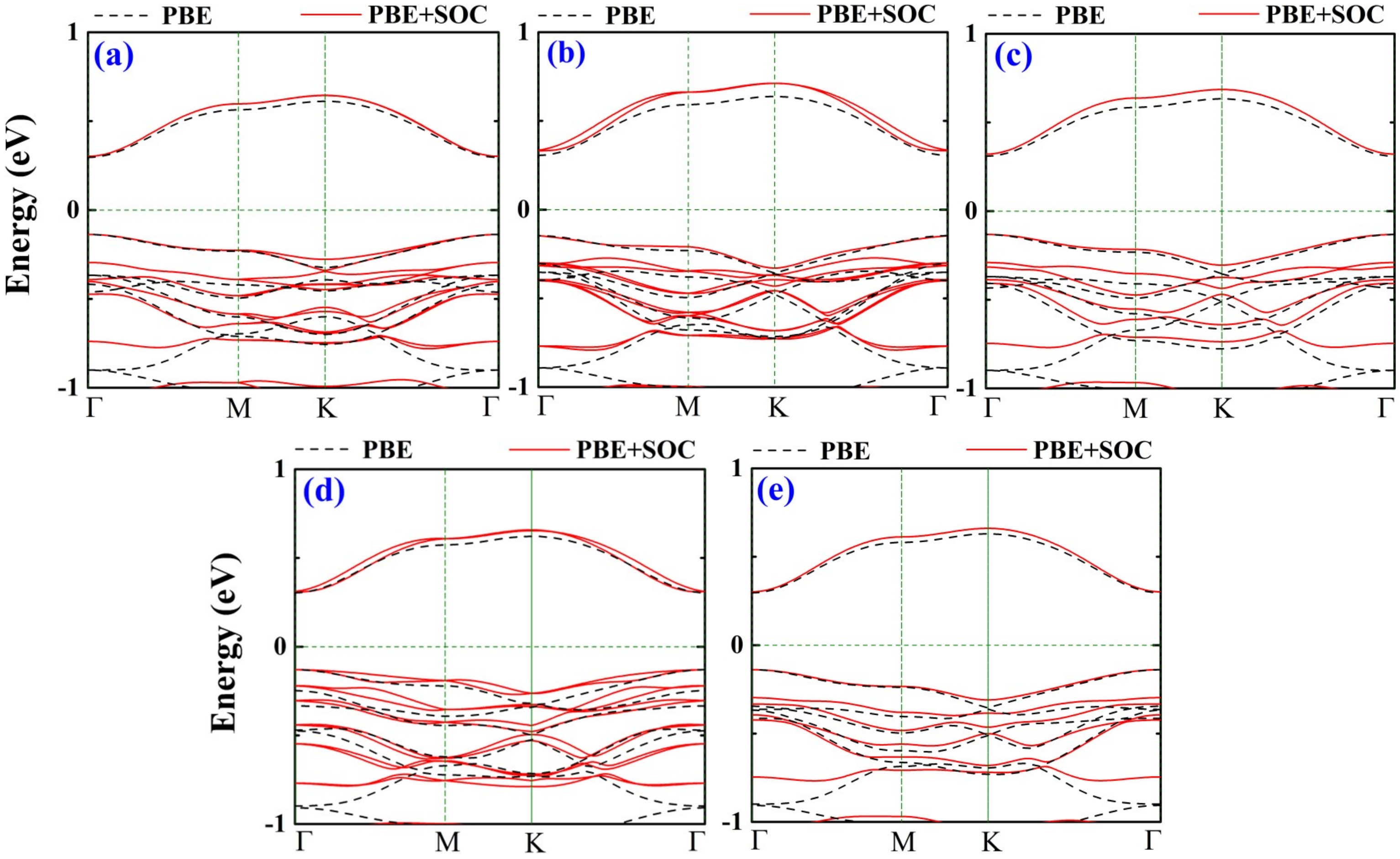

Fig. S5. Band structure of (a) *P6m2*, (b) *P6mm*, (c) *P3m1*, (d) *Cmm2,* and (e) *C2/m*-$W_8S_6Se_6$ monolayers. Black dashed lines: PBE calculations; red solid lines: PBE+SOC calculations with spin–orbit coupling.

Table SI. Calculated band gaps obtained at the PBE and PBE+SOC levels for *P*6*m*2, *P*6*mm*, *P*3*m*1, *Cmm*2, and *C*2/*m*-$W_8S_6Se_6$ monolayers.

$W_8S_6Se_6$	*P6m2*	*P6mm*	*P3m1*	*Cmm2*	*C2/m*
Eg (PBE)/eV	0.43	0.45	0.44	0.43	0.43
Eg (PBE+SOC)/eV	0.44	0.47	0.45	0.44	0.44

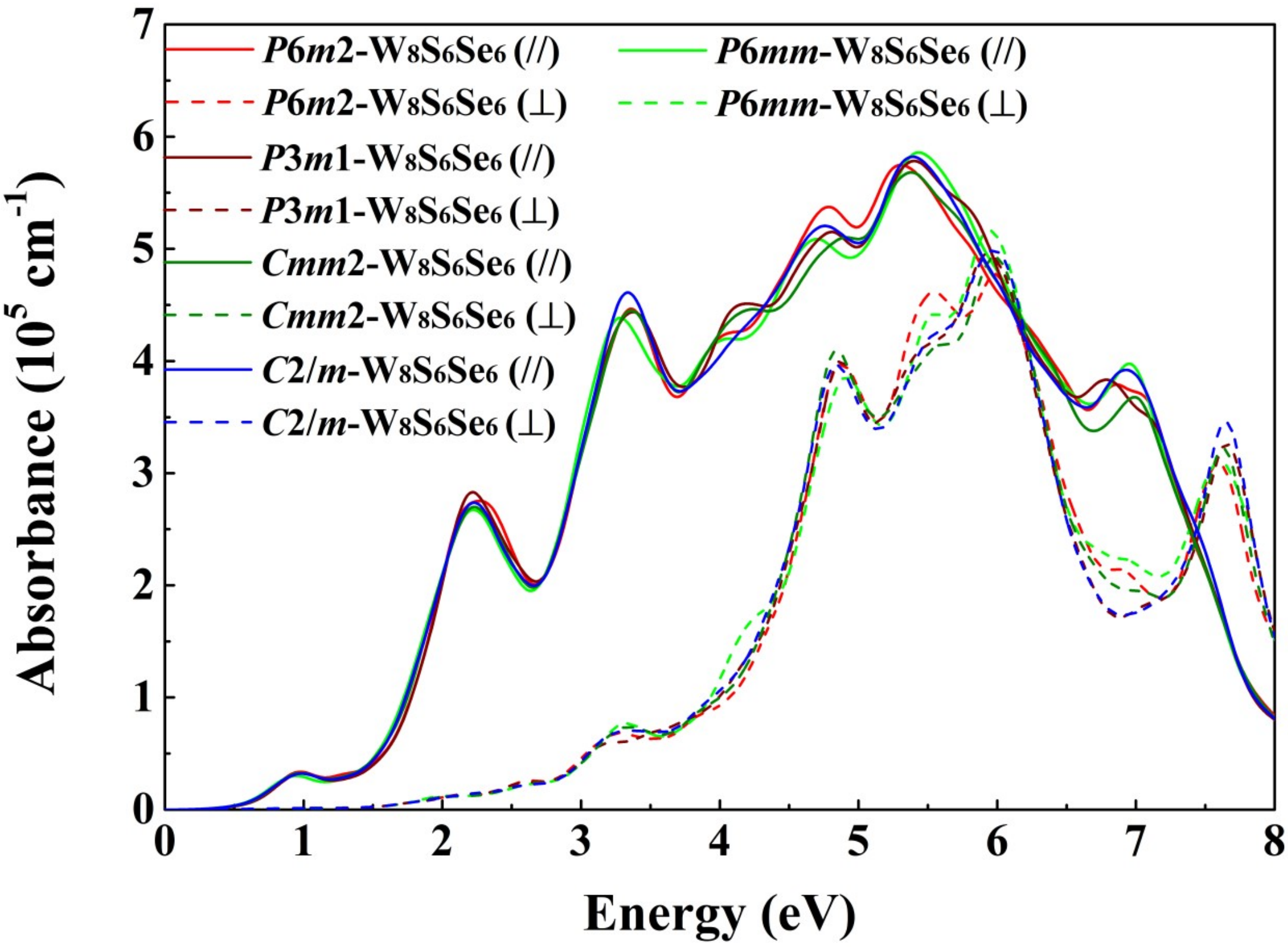

Fig. S6. PBE-calculated anisotropic absorption coefficients for in-plane (//) and out-of-plane (⊥) polarizations of *P6m2*, *P6mm*, *P3m1*, *Cmm2,* and *C2/m*-$W_8S_6Se_6$ monolayers.

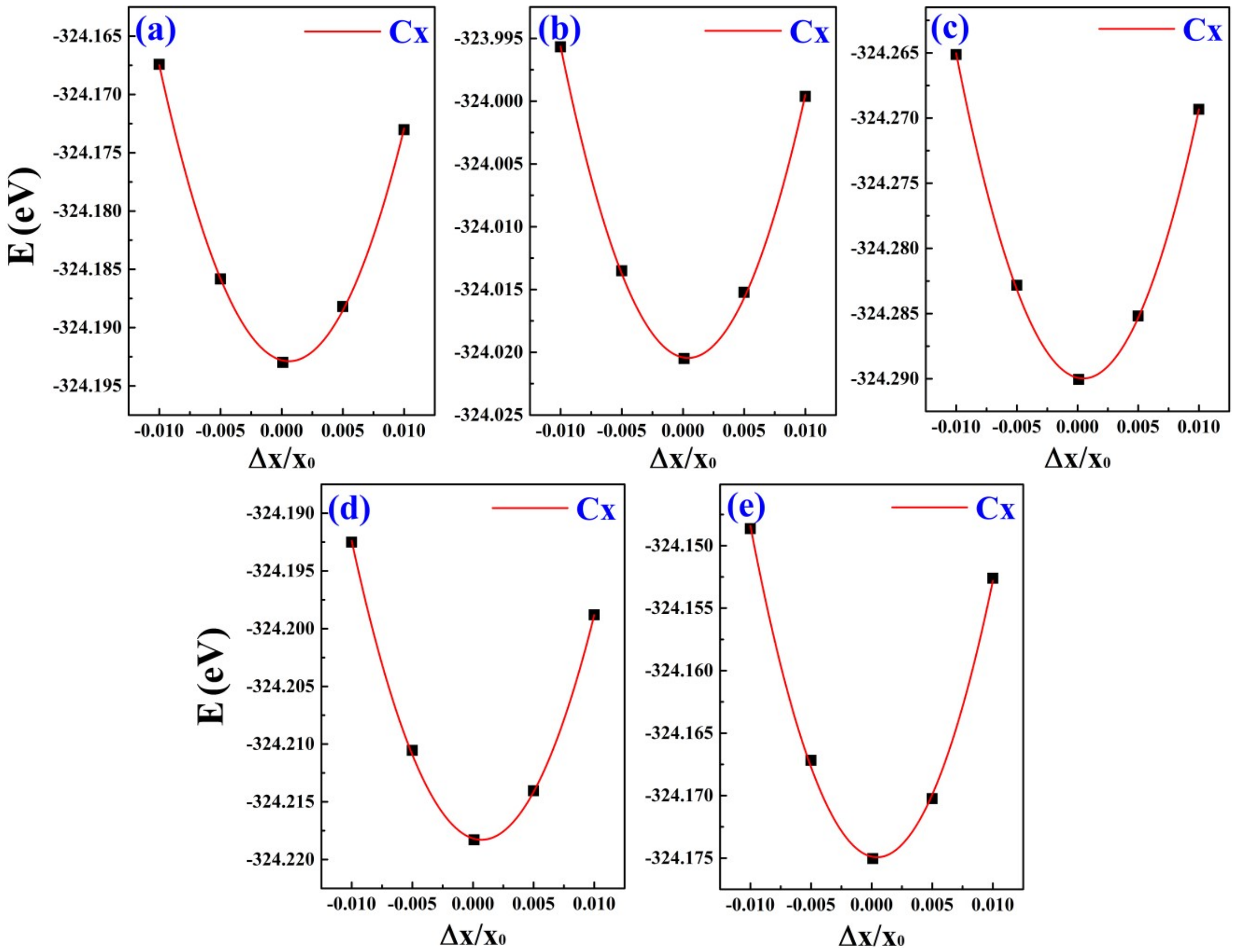

Fig. S7. The relationship between the total energy of (a) *P6m2*, (b) *P6mm*, (c) *P3m1*, (d) *Cmm2, and* (e) *C2/m*-$W_8S_6Se_6$ monolayers under uniaxial strain and the strain along the x direction, respectively. The in-plane stiffness *C* can be obtained after fitting the parabola.

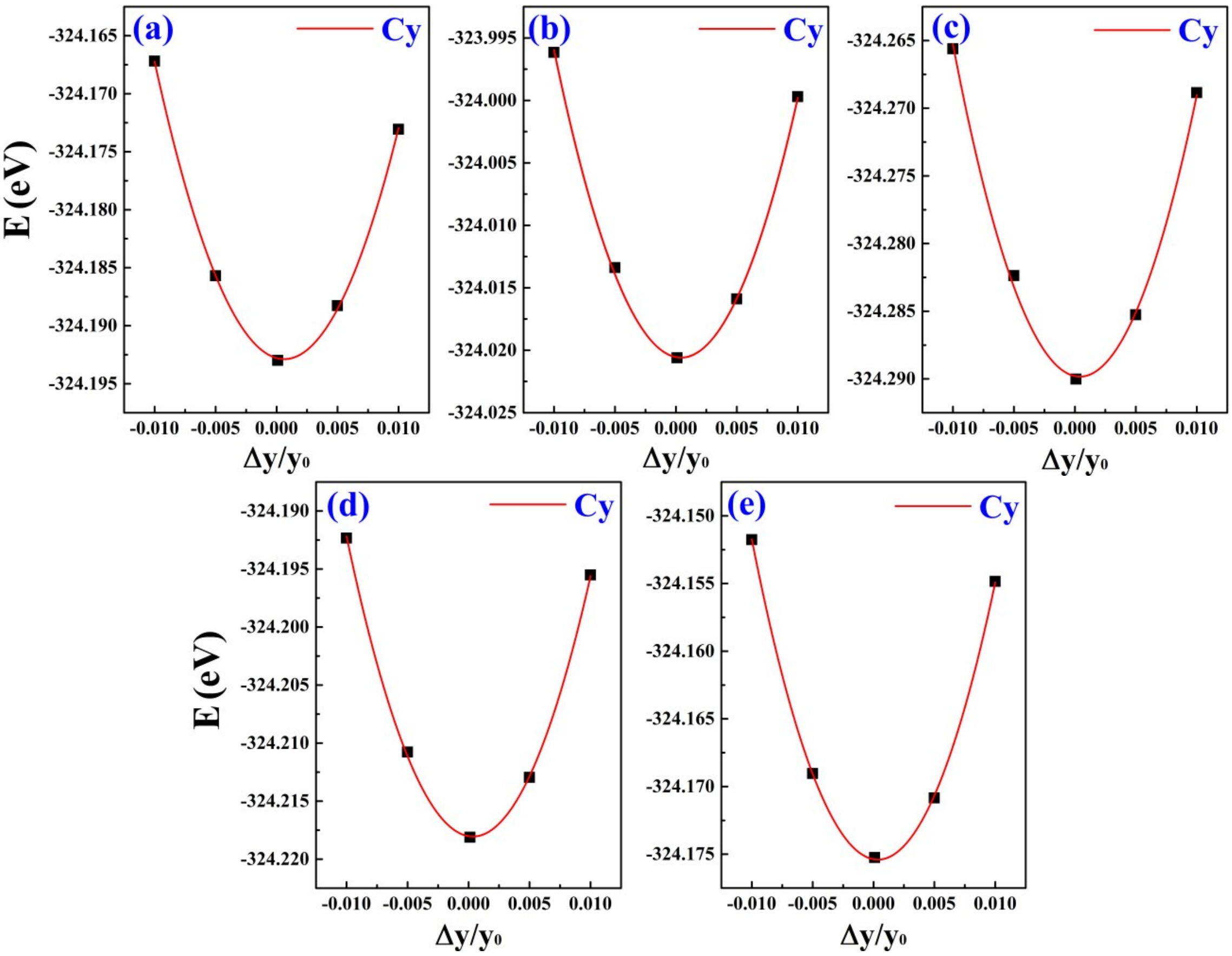

Fig. S8. The relationship between the total energy of (a) *P6m2*, (b) *P6mm*, (c) *P3m1*, (d) *Cmm2, and* (e) *C2/m*-$W_8S_6Se_6$ monolayers under uniaxial strain and the strain along the y direction, respectively. The in-plane stiffness *C* can be obtained after fitting the parabola.

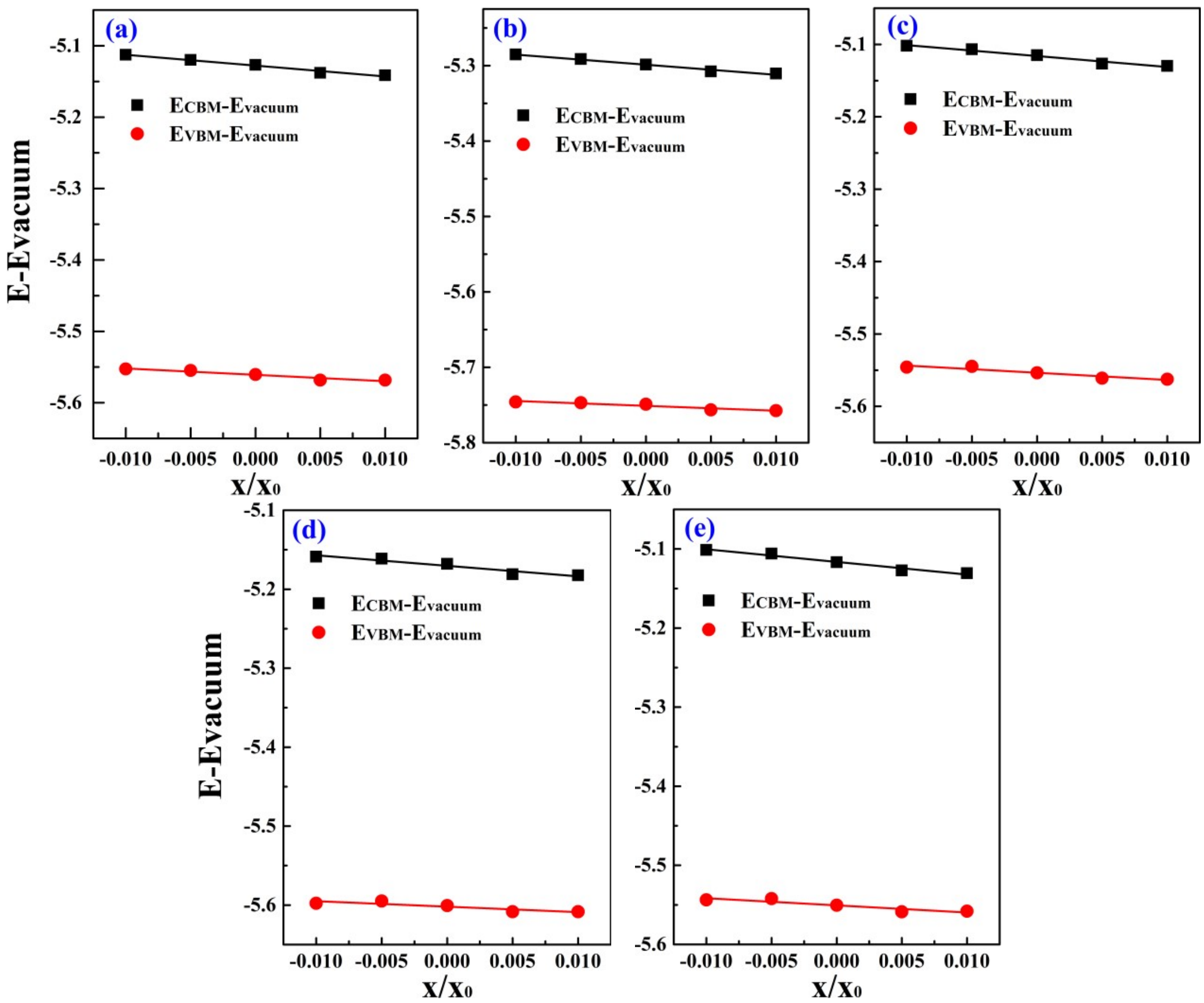

Fig. S9. The energy difference between the band energies of the CBMs and VBMs relative to the vacuum energy in (a) *P6m2*, (b) *P6mm*, (c) *P3m1*, (d) *Cmm2, and* (e) *C2/m*-$W_8S_6Se_6$ monolayers is illustrated as a function of lattice expansion along the x direction. The gradients of the black and red lines correspond to the DP constant in their respective directions for electrons and holes.

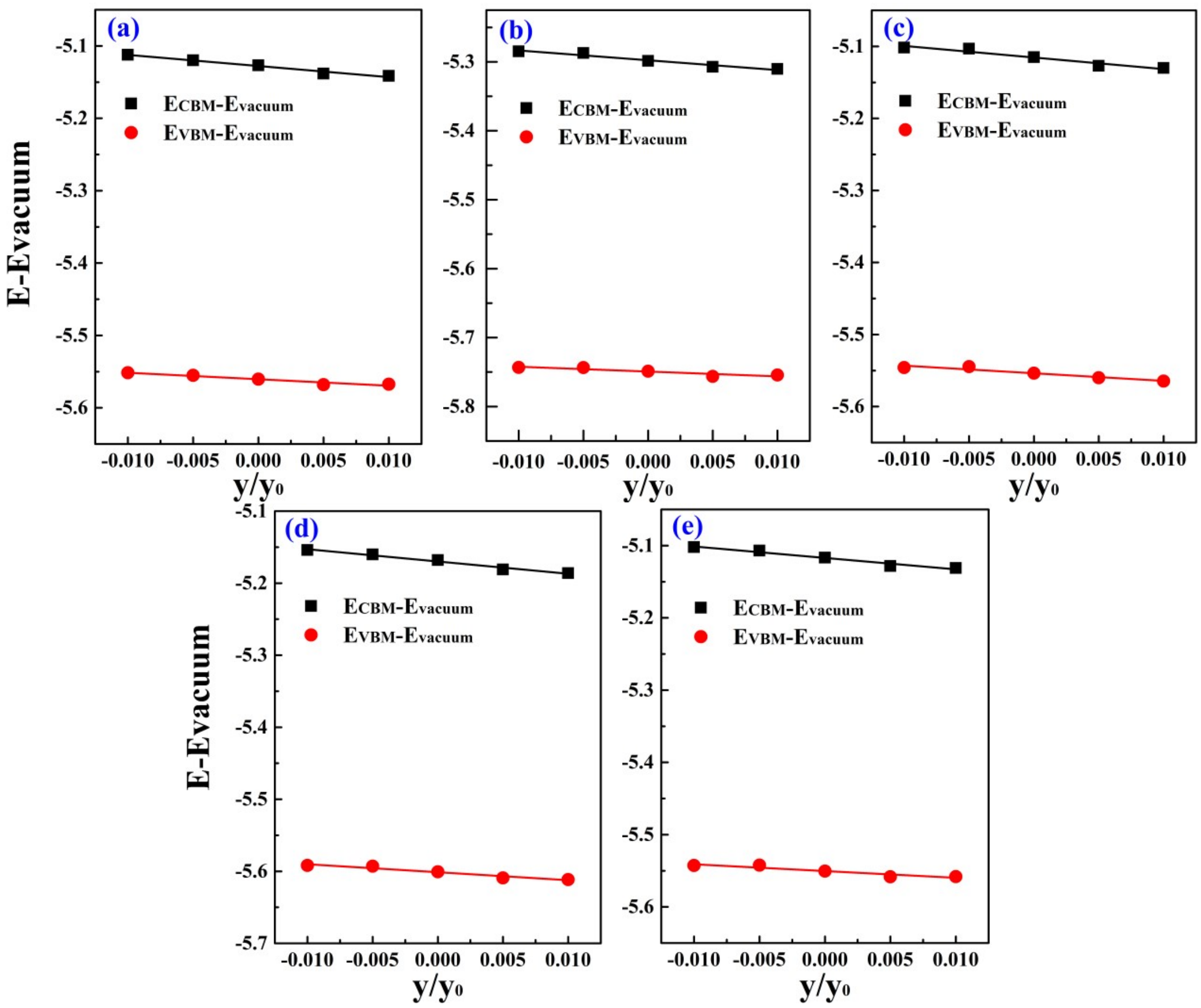

Fig. S10. The energy difference between the band energies of the CBMs and VBMs relative to the vacuum energy in (a) *P6m2*, (b) *P6mm*, (c) *P3m1*, (d) *Cmm2, and* (e) *C2/m*-$W_8S_6Se_6$ monolayers is illustrated as a function of lattice expansion along the y direction. The gradients of the black and red lines correspond to the DP constant in their respective directions for electrons and holes.

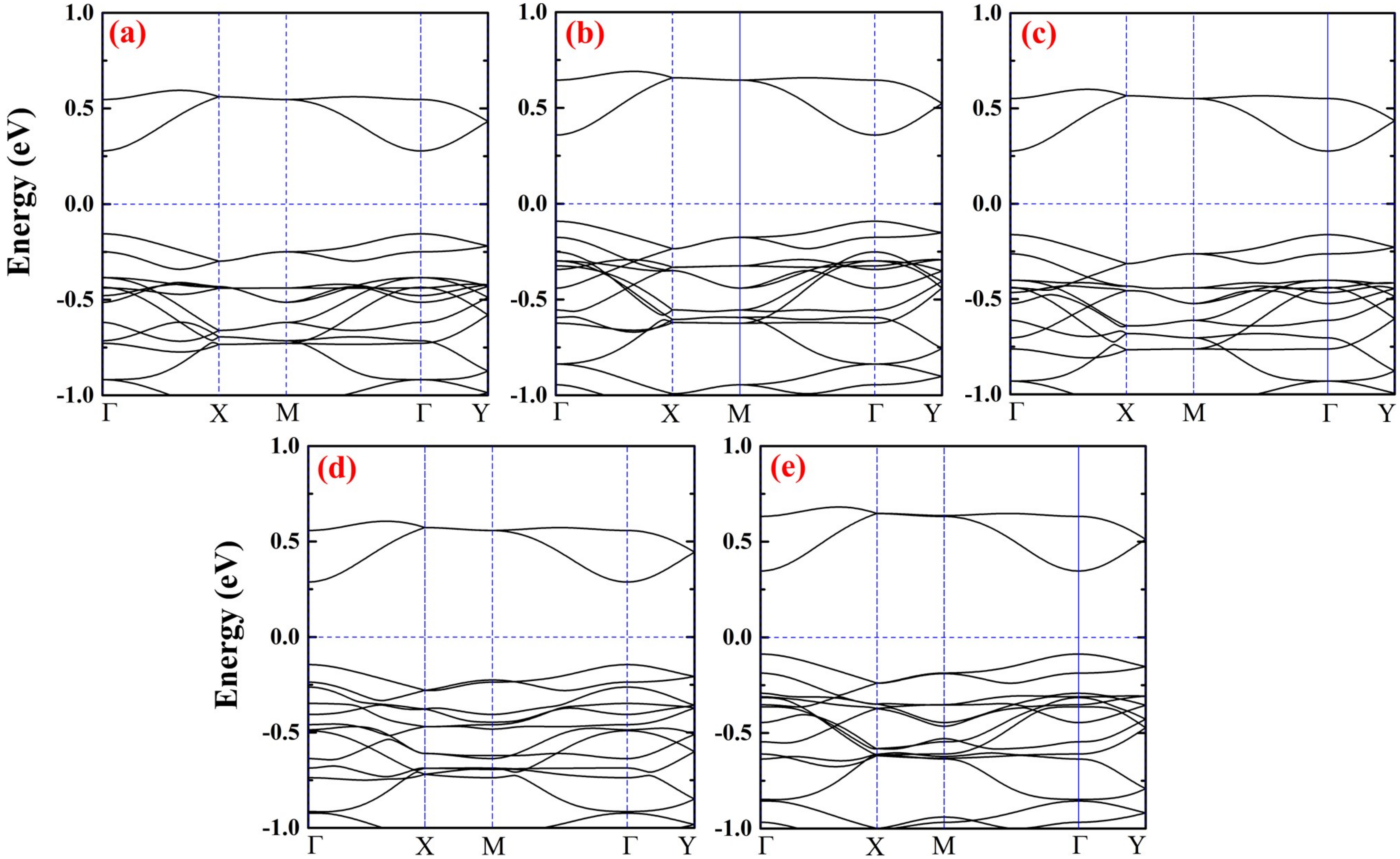

Fig. S11. Band structures of (a) *P6m2*, (b) *P6mm*, (c) *P3m1*, (d) *Cmm2,* and (e) *C2/m*-$W_8S_6Se_6$ monolayers calculated along the orthogonal Brillouin zone path.
